# Synovial Fibroblast Sialylation Regulates Cell Migration and Activation of Inflammatory Pathways in Arthritogenesis

**DOI:** 10.3389/fimmu.2022.847581

**Published:** 2022-03-18

**Authors:** Yilin Wang, Piaopiao Pan, Aneesah Khan, Çağlar Çil, Miguel A. Pineda

**Affiliations:** ^1^Institute of Infection, Immunity and Inflammation, University of Glasgow, Glasgow, United Kingdom; ^2^Research Into Inflammatory Arthritis Centre Versus Arthritis (RACE), Glasgow, United Kingdom

**Keywords:** synovial fibroblast (FLS), sialic acid, glycoimmunology, rheumatoid arthritis, inflammation, migration, cytokines

## Abstract

Synovial fibroblasts have emerged as critical underlying factors to perpetuate chronic joint inflammation in Rheumatoid Arthritis. Like any other cell, synovial fibroblasts are covered with a complex layer of glycans that can change in response to extracellular signals, such as inflammation. We have previously shown that inflammatory synovial fibroblasts show decreased levels of sialic acid, but our understanding of sialic acid-dependent pathophysiological pathways in these stromal cells is still very limited. In this report, we used *in vivo* and *in vitro* studies with exogenous sialidases and RNA sequencing to investigate the responses of murine synovial fibroblasts upon desialylation. Our results show that hyposialylated fibroblasts present a dysregulated migratory ability and an activated phenotype characterized by the expression of inflammatory mediators, such as cytokines and chemokines, and anti-viral related mechanisms. Removal of surface sialic acid also affected the expression of sialyltransferases, revealing the existence of a positive feedback to sustain reduced sialylation. Moreover, we demonstrate that synovial fibroblasts subsets have distinct sialyltransferase expression profiles, both in healthy and arthritic mice. These findings underline the ability of sialic acid to modulate homeostatic and inflammatory responses in non-immune synovial fibroblasts, suggesting that sialylation plays a key role in perpetuating local inflammation in the arthritic joint.

## Introduction

Rheumatoid Arthritis (RA) is an autoimmune joint condition that causes pain, swelling and stiffness in the joints, the result of an ongoing chronic inflammatory process. Inflammation is however an essential defensive instrument of the human body, starting protective responses and subsequent healing processes to restore tissue homeostasis. Dysregulation of these mechanisms lead to a persistent inflammatory phenotype characteristic of chronic RA, whose primary target is the joint synovium, the soft tissue that lines the inner surfaces of diarthrodial joints. In health, this membrane nourishes the cartilage and bone tissue. Nonetheless, the synovial membrane becomes noticeably thicker in RA promoting immune cell infiltration and cartilage and bone damage (1). Genetic, epigenetic, and environmental factors can contribute to disease initiation (2) but specific mechanisms are still unclear. Equally importantly, we still do not fully understand why inflammation persists in RA, although recent research in the field of stromal immunology has shown that non-immune cells such as Synovial Fibroblasts (SFs) play a critical role in perpetuation of RA. SFs are a basic part of the synovium and they become activated during inflammatory arthritis, secreting cytokines, such as IL-6, GM-CSF and chemokines like Cxcl10, Ccl2, IL-8 that attract macrophages, neutrophils and lymphocytes (3–6). SFs also support ectopic tertiary lymphoid structures to continue aberrant immune responses in the joint (7). These recruited immune cells increase the local concentration of TNFα, IL-1β or IL-17, that continue to promote SF activation (8–10) generating pathogenic self-perpetuating inflammatory loops. Furthermore, SFs become hyperproliferative, migrate to bone and cartilage inducing tissue damage, secrete matrix-degrading enzymes and RANKL and promote local angiogenesis upon VEGF secretion (11–13). Interestingly, recent findings based on single cell RNA sequencing have demonstrated the existence of distinct SFs subsets with specific anatomical location within the synovium and non-overlapping effector functions (14, 15). For example, FAPα+CD90+ SFs found in the sublining synovium lead the immune effector function, whereas lining FAPα+CD90− SFs drive cartilage destruction (16). Nevertheless, there is still a lack of clinical targets to intervene SFs in the clinic.

Loss of inhibitory or regulatory signalling is a critical mechanism to trigger autoimmunity and chronic inflammation. At the heart of many of these signals is the cell glycome, which comprises the entire pool of glycans found at the cell-cell interface. The outermost monosaccharide decorating glycans in humans is usually a molecule belonging to the Sialic Acid (SA) family. Given its location, negative charge and hydrophilicity, SA modulates a wide variety of pathological processes. An increasing body of evidence supports the hypothesis that SA acts an immune check-point (17), as high SA levels can deliver anti-inflammatory or tolerogenic signals, whereas low concentration of SA is linked to inflammation. Corroborating this theory, multiple cancer cells overexpress α2,3, α2,6, and α2,8 linked SA to evade immune responses, inhibiting NK, T and B cells *via* SA-Siglec signalling (18). On the other hand, deficiency of CD22 (Siglec-2) and Siglec G leads to hyperactivated B cells and autoimmunity (19, 20), including exacerbation of experimental arthritis and lupus (21). Besides, activation of TLR-NFκB-mediated responses in immune cells appears to be associated with a reduction of SA on the cell surface upon sialidase activity (22–25). This agrees with our previous results, where we have shown that TNFα-mediated down-regulation of α2-6 sialylation is a hallmark of activated SFs in experimental arthritis (26). In this report, we first describe the sialylation pathways associated to distinct SFs subsets in healthy and arthritic mice. Second, we investigate the responses of murine SFs upon *in vitro* cell surface desialylation, hypothesising that enzymatic removal of sialic acid in healthy SFs cell surface would trigger intracellular signalling to initiate inflammatory responses. Our data indicate that desialylated SFs rapidly adopt a phenotype reminiscent of the SFs found in the arthritic joint, characterised by enhanced cell migration, activation of NFkB-mediated pathways and antiviral responses. Interestingly, removal of SA also modulates mRNA expression of the sialyltransferases ST6Gal1 and ST3Gal3, suggesting that environmental or temporal changes affecting SA content may be perpetuated in time to consolidate local inflammation.

## Methods

### *Ex Vivo* Culture of SFs

Isolation and *ex vivo* expansion of murine SFs was done as previously described (27). Briefly, paws were harvested from mice, skin and soft tissue were removed, synovial tissue was dissected and digested with type II collagenase (1 mg/ml; Sigma #C6885) for 80 minutes at 37°C. Samples were vortexed vigorously to release cells and centrifuged. For *ex vivo* expansion, cells were resuspended in DMEM (#21969-035) supplemented with 10% fetal calf serum (FCS; #10270106), 1% penicillin and streptavidin (#15140122), 1% L-glutamine (#25030-024) and 1% NEAA (#11140-035, all from Invitrogen, UK) and cultured in 5% CO2 at 37°C for 24 hours, when culture medium was replaced. Media was changed twice a week and the cells were passaged at 90% confluence using trypsin-EDTA (Invitrogen, #25300-054). Prior to experimental setup, expression of Podoplanin (PDPN, Biolegend, #156204) and CD11b (Invitrogen, #11-0112-85) was assessed by flow cytometry. Myeloid CD11b+ cells were labelled with biotinylated anti-CD11b antibody (Biolegend, #101204) and subsequently depleted using Streptavidin magnetic MicroBeads (MACS Miltenyi Biotec, #130-090-485). For *in vitro* cytokine stimulation, recombinant IL-1β was used at the indicated concentrations for 6 hours in cDMEM.

### Collagen-Induced Arthritis (CIA) Mouse Model

8-10 weeks male DBA/1 mice were purchased from Envigo (UK) and maintained in the Biological Services Unit of University of Glasgow in according to the Ethics Review Boards (AWERB) of University of Glasgow and the Home Office UK licences PIL IF5AC4409 and PPL P8C60C865. Mice received 100 μg of chicken type II collagen (MD Bioproducts #804002-Sol) emulsified with an equal amount of complete Freund’s adjuvant (CFA, MD Bioproducts #501009) on day 0 *via* intradermal injection above the tail base. On day 21, mice were injected intraperitoneally with 100 μg collagen in PBS. Mice were monitored every two days for body weight, paw thickness and clinical scores. Clinical scores were assigned according to clinical signs, using a scale from 0 to 4 for each paw. An overall score exceeding 10 or weight loss exceeding 20%, paw thickness exceeding 4.5 mm or more than three inflamed paws was considered as an experimental endpoint and the mouse was immediately euthanized.

### FACS Sorting of SFs Subsets

Cells from mouse synovium were obtained as described above for cell culture, with the addition of DNase I (1 mg/ml; Sigma #DN25) during collagenase digestion. Cells were then resuspended in red cell lysis buffer for 3 min at room temperature, and red cell lysis was stopped by adding 20 ml of cold PBS. Cells were then centrifuged and stained with flexible viability dye eFluor 780 (Invitrogen #65-0865-14) at 1 μg/ml in PBS for 20 min on ice to discriminate live and dead cells. FC receptor was blocked using CD16/CD32 specific antibody (Invitrogen, #14-0161-85) for 20 min on ice. Cells were then incubated with primary antibodies or isotype controls at 1 μg/ml in FACs buffer (PBS 1%FBS 2 mM EDTA) for 20 min at 4°C. Antibodies used were: anti-CD31PE (Invitrogen, #12-0311-81), anti-CD45-PE (Biolegend, #103106), anti-CD90-FITC (Biolegend, #105316), anti-PDPN-A647 (Biolegend, #156204), anti-rat IgG2b-PE (BD bioscience #25393), anti-rat IgG2a-PE (Biolgend, # 400508), anti-rat IgG2b-FITC (Biolegend #400634) and anti-rat IgG2a-APC (Biolegend, #400512). Cell sorting was performed using FACS Aria III or FACS Aria IIu (all from BD), data were analyzed with FlowJo software 10.7.1.

### Desialylation of Synovial Fibroblasts *In Vitro*

To hydrolyse sialic acid, SFs were cultured until reaching 90% confluence. Cells were washed three times with cold PBS and incubated for 1 hour with 100 mU Neuraminidase from Clostridium perfringens (CP, Roche, #11585886001) diluted in sialidase buffer (PBS: RPMI 1640 = 1:1, pH=6.8) or with sialidase buffer only (negative control, NT). Cells were washed with cold PBS prior to assessing the removal of sialic acid by lectin staining, or washed and cultured with cDMEM 10% FCS for further RNA extraction. RNA was isolated as explained below, and used for RNA-Seq or RT-PCR experiments.

### RNA Isolation and RT-qPCR

RNA from SFs was isolated using either RNeasy Micro Kit (Qiagen #74004) or EZ-10 RNA Mini-Preps Kit (Bio Basic #BS88136) according to the manufacturer’s instructions. Reverse transcription was performed using High-Capacity cDNA Reverse Transcription Kit (Thermo Fisher Scientific #4368814) and RT-qPCR was performed using TaqMan™ Gene Expression Assay (Applied Biosystem). The expression of actin mRNA was used as an endogenous control to normalise samples. Taqman predesigned primers (Applied Biosystem) were: Actb/Mm02619580_g1; IL-6/Mm00446190_m1; CCL2/Mm00441242_m1; MMP3/Mm00440295_m1; MMP13/Mm00439491_m1; TNFRSF11b/Mm00435454_m1; TNFSF11/Mm0041906_m1; St6gal1/Mm00486119_m1; St6galnac5/Mm00488855_m1, St3gal1/Mm00501493_m1; St3gal2/Mm00486123_m1; St3gal3/Mm00493353_m1; St3gal4/Mm00501503_m1, St3gal6/Mm00450674_m1; Myd88/Mm00440338_m1 and NFKBIB/Mm01179097_m1.

### Cell Migration Assay

SFs (10^4^) were seeded in u-dishes (ibidi, #80466) coated with fibronectin (R&D systems #1030-FN) until reaching 90% confluence. U-dishes have a plastic insert that leaves a cell-free gap when removed. Cells were allowed to grow in the gaps after insert removal, and cell-free areas were measured after gap was created (T0) and 24 hours later (T24). Following monitoring of cell cultures, 24 hours was selected as our experimental time point because it allowed sufficient cell migration to observe biological differences without completely covering the cell-free region. Cell migration was quantified by measuring the difference in the width of cell-free region between T0 and T24, calculated with ImageJ software.

### Flow Cytometry

For proliferation studies, cells were labelled with 10 μM proliferation dye eFlour 670 (eBiosciences, #65-0840-90) for 10 min on ice. Labelling was stopped by adding 4-5 volumes of complete culture medium for 5 min on ice. Cells were then subjected to flow cytometry analysis (day0) or maintained in culture for additional 5 days prior to analysis by Flow Cytometry. For lectin staining, Peanut Agglutinin (PNA, #B-1075), Sambucus Nigra Lectin (SNA, #B-1305), Aleuria Aurantia Lectin (AAL, #B-1395) and Maackia Amurensis Lectin II (MAA, # B-1265-1), all from vector laboratories, were used. Cells were blocked in carbon-free blocking buffer (vector laboratories, #SP-5040) for 20 min on ice, and then incubated with biotinylated lectins diluted in PBS containing 5% carbon-free blocking buffer. Lectins were then detected with FITC-conjugated streptavidin (Biolegend, #405201), Alexa Flour 647-conjugated streptavidin (Biolegend, #2068269) or PE-conjugated Streptavidin (Biolegend, #410504) in PBS for 20 min at 4°C. To differentiate between live and dead cells, all samples were stained with DAPI (Sigma, #32670, dilution 1:1000) prior to data acquisition. Data were acquired using an LSR II flow cytometer (BD) and analysed using FlowJo version 10.8.0.

### RNA-Sequencing (RNA-Seq) and Data Analysis

Total RNA from cultured SFs was isolated, RNA integrity check was performed using the Agilent 2100 Bioanalyzer System and RNA integrity number (RIN) value was > 9 for all samples. Library preparation was done using RNA poly A selection at Glasgow Polyomics (Glasgow, UK). Low sequencing reads were removed using Trimmomatic (28) before mapped to mouse reference genome (GRCM38) using Hisat2 version 2.1.0. Featurecounts version 1.4.6 was used to quantify reads counts. Mouse ENSEMBL gene ID to gene symbol conversion was performed in BioTools (https://www.biotools.fr). Differentially expressed (DE) genes were identified using DESeq2, and Principal component analysis (PCA) were performed using R Bioconductor project DEbrowser (29). Genes passing a threshold of Padj<0.01 and |log2Foldchange| > 1 were considered as differentially expressed. Gene Ontology (GO) Biological Process enrichment and KEGG pathway enrichment were conducted with Metascape (30) and PathfindR (31).

### MTS Assay Protocol

The MTS assay kit (Abcam, # ab197010) was used to measure the cellular metabolic activity of SFs according to manufacturer’s instructions. Briefly, cells were grown in 96-well plates (10,000 cells/well), medium was removed and 100 μL of cDMEM and 10 μL of MTS solution were added into each well. Plates were incubated for 4 hours at 37°C when absorbance was read at optical density of 590 nm. The same amount of cDMEM and MTS solution without cells was used as an internal control for no metabolomic changes and background absorbance.

### Statistical Analysis

Data are presented as the mean ± standard error (SEM). Statistical analysis was performed using Prism 8 software (GraphPad). One-way analysis of variance (ANOVA) was used to test significant differences among multi-groups, and student t-test was used between two groups studies. P values <0.05 were considered significant.

## Results

### SFs Subsets Show Distinctive Regulation of Sialyltransferases in Arthritic Mice

**Figure 1:**
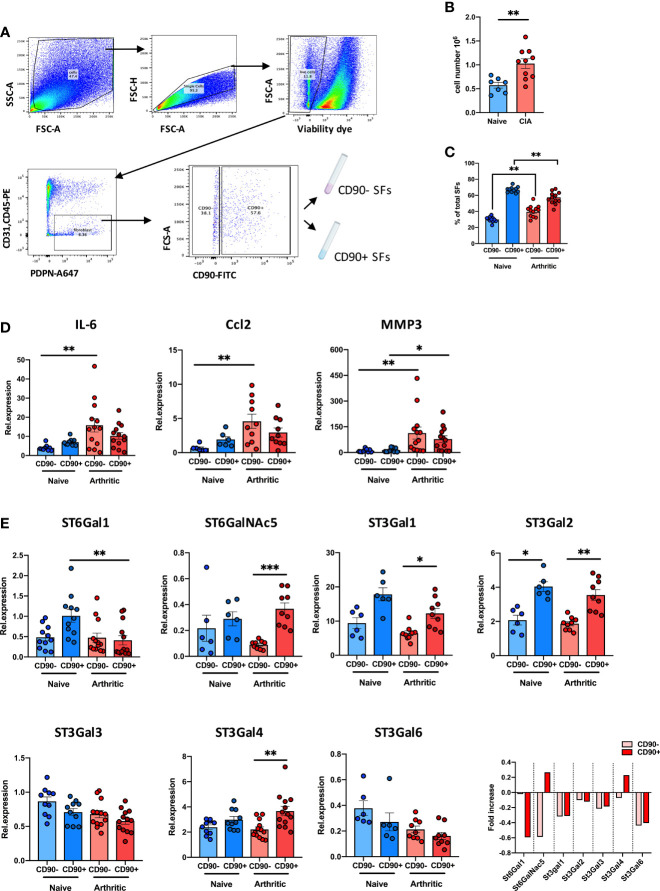
CD90+ SFs show a distinct expression of sialyltransferases in experimental arthritis. **(A)** SFs were isolated from joints of naïve and mice undergoing Collagen-Induced Arthritis. Cells were sorted by FACs gating on low viability dye, CD31-, CD45- and PDPN+ cells. Subsets of SFs were discriminated by expression of CD90. Sorting strategy is shown in the dot plots. **(B)** Total number of isolated cells from paws from naïve (n = 7) and CIA (n = 10) animals. **(C)** Relative proportions of SFs subset (lining: CD90- and sublining: CD90+) in total SFs of naïve (n = 11) and arthritic mice (n = 12) evaluated by flow cytometry. **(D)** Relative expression of IL-6, CCL2 and MMP3 mRNA in SFs subsets were quantified by RT-qPCR (n ≥ 6). **(E)** Relative expression of α2,3- and α2,6-sialyltransferases (St6gal1, St6GalNac5, St3gal1, St3gal2, St3gal3, St3gal4 and St3gal6) were quantified in SFs subsets by RT-PCR (n ≥ 6). Data are represented as mean ± SEM; each dot represents SFs from one individual mouse. **p < 0.01 by Mann-Whitney test in **(B, C)**, *p < 0.05, **p < 0.01, ***p < 0.001 by Kruskal-Wallis test in **(D, E)**.

We have recently shown that inflammatory SFs isolated from mice undergoing experimental Collagen-Induced Arthritis (CIA) show reduced expression of the glycosyltransferase ST6Gal1 and associated α2-6 sialylation (26) but we did not have data about the relative expression of other sialyltransferases in SFs subsets. Therefore, to continue investigating SFs sialylation profile in joint disease, we isolated SFs from healthy and arthritic CIA mice. SFs were identified by flow cytometry as podoplanin+ and CD45-CD31- (to exclude immune and endothelial cells), and expression of CD90 was used to sort lining (CD90-) and sublining (CD90+) SFs (**Figure 1A**), subsets that have shown distinct anatomical locations and pathophysiological roles (14). As expected, we recovered a higher number of cells from the arthritic joints (**Figure 1B**) and the relative proportion of CD90- versus CD90+ SFs was altered (**Figure 1C**). RNA was extracted from naïve and CIA sorted CD90- and CD90+ SFs, and expression of IL-6, Ccl2 and MMP3 was evaluated by RT-PCR (**Figure 1D**). CIA SFs showed elevated expression of these inflammatory markers, corroborating their activated/inflammatory status compared to healthy SFs. In line with previous reports, SFs subsets showed a differential expression pattern for inflammatory cytokines IL-6 and Ccl2, with a more noticeable distinction under non-inflammatory conditions. Next, we quantified the mRNA expression of ST6Gal1, ST3Gal1, ST3Gal2, ST3Gal3, ST3Gal4, ST3Gal6 and ST6GalNAc5, sialyltransferases expressed in murine SFs involved in glycoprotein sialylation (**Figure 1E**). SFs subsets showed a different sialyltransferase expression profile, probably reflecting their different biological role and anatomical location. In healthy synovium, CD90+ SFs presented higher expression of ST6Gal1, ST3Gal1 and ST3Gal2 compared to CD90- cells. However, in inflammatory CIA conditions, CD90+ cells down-regulated expression of ST6Gal1 (which adds α2-6-linked sialic acids to glycoproteins), whereas enzymes involved in α2-3 sialylation remained unaltered or even up-regulated in the case of ST3Gal4. These results corroborate our previous findings (26) and also provide further support to the hypothesis that reduced α2-6 sialylation is an inflammatory checkpoint in CD90+ SFs.

### Enzymatic Removal of Sialic Acid Enhances SF Migration

**Figure 2:**
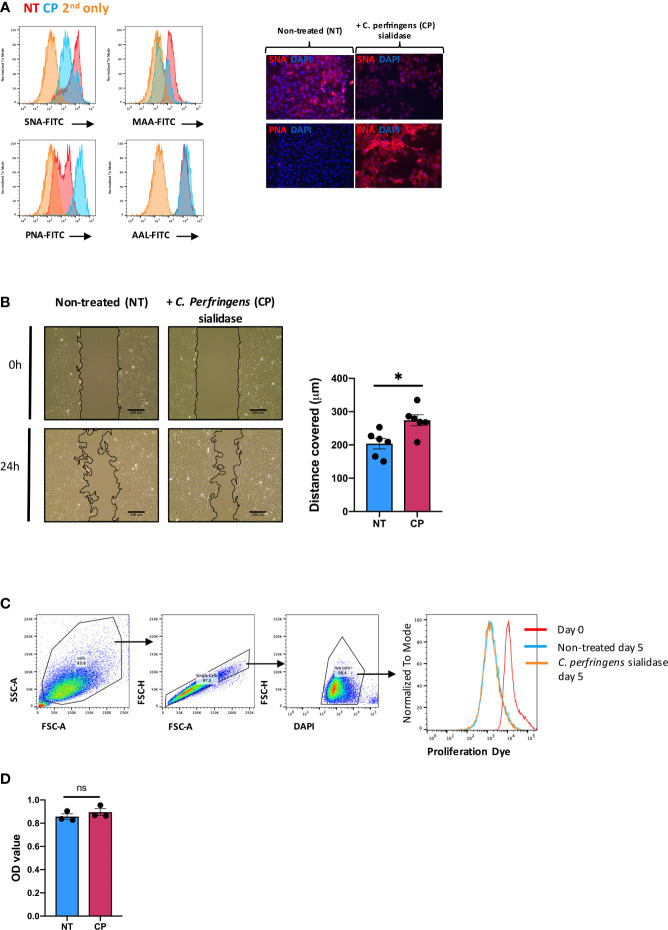
*In vitro* desialylation enhances SFs motility. SFs isolated from naïve mice were cultured and expanded *ex vivo*. Cells were cultured to reach confluency and then treated with 0.1U/ml of *C. perfringens* sialidase (CP) for 1 hour to remove sialic acid from cell surface. **(A)** Control and sialidase-treated cells were stained with biotinylated lectins (SNA, MAA, PNA and AAL), followed by incubation with Alexa-647 streptavidin. Cells were then examined by flow cytometry to quantify lectin binding, as shown in histograms. Images were acquired in an EVOS™ FL Auto microscope, showing SNA and PNA-stained cells. Error bars: 200 μm. **(B)** SFs were seeded in migration chambers and grown until monolayer confluence to conduct migration assays. Images show one representative experiment, including control and sialidase-treated naïve SFs when insert was removed (time 0, T0) and 24 hours after desialylation (T24). Superimposed black lines define the cell-free area, scale bar: 200 μm. Column graph show the mean migration distance of 5 independent experiments, showing non-treated (NT) and sialidase-treated (CP) cells. Error bars represent SEM, each dot represents one independent experiment (n = 5), *p < 0.05 by Mann-Whitney test. **(C)** SFs were labelled with proliferation dye eFluor 670 and analysed directly by flow cytometry (Day0) or treated with sialidase and maintained in culture for 5 days (Day5). Histogram shows one representative experiment. **(D)** Naïve SFs were seeded in 96-well plates, treated with sialidase and maintained in culture condition to assess cell viability and metabolomic activity using a colorimetric MTS assay. Each dot represents one independent experiment (n = 3) analysed in technical triplicate, error bars represent SEM, ns, non-significant, by Mann-Whitney test.

Next, we sought to examine the pathophysiological function(s) of SA in SFs. To this end, cells were expanded *ex vivo* from healthy murine synovium, as cultured SFs maintain most of their epigenetic and phenotypic signatures for several passages (32, 33). Furthermore, cultured SFs express high and homogeneous levels of CD90 (9). Such phenotype is reminiscent of sublining SFs, subset that shows down-regulated ST6Gal1 expression during joint inflammation (**Figure 1E**). Thus, expanded SFs provided a suitable tool for *in vitro* experiments in this context. To mimic the desialylation observed *in vivo* during disease, we treated SFs with *Clostridium perfringens* sialidase. Sialidase treatment reduced levels of both α2-6- and α2-3-linked SA as evidenced by the reduced binding of *Sambucus nigra* agglutinin (SNA) and *Mackia amurensis* agglutinin (MAA) (**Figure 2A**). Cells showed 49.4% ( ± 0.029) SNA binding and 25.3% ( ± 0.038) MAA binding after desialylation. The sialidase specificity was further confirmed by an increased Peanut agglutinin (PNA) binding, since the presence of SA inhibits its glycan recognition, and unaffected binding of *Aleuria aurantia* agglutinin (AAL), a fucose specific lectin (**Figure 2A**). SA has been linked to cell migration, with reports showing that both α2-6 and α2-3-linked sialic acid can promote cell migration in various cell types and cancer (34–37). Because SFs adopt a migratory phenotype during RA, we hypothesized that SFs with reduced levels of SA would have an increased migration capacity, similar to the activated cells in RA. To test this, SFs were desialylated with *C. perfringens* sialidase and cell migration was evaluated using wound healing assays on fibronectin coated wells. Results confirmed the proposed hypothesis, since cell migration was significantly increased in desialylated SFs (**Figure 2B**). By contrast, neither cell proliferation (**Figure 2C**), nor cellular metabolomic rate (**Figure 2D**) were affected.

### SA Removal Induces Rapid Pro-Inflammatory Transcriptomic Changes in Healthy SFs

**Figure 3:**
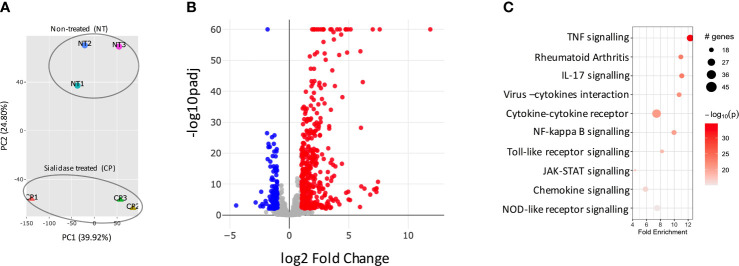
Sialidase-treated SFs show an inflammatory transcriptomic profile. SFs from naive mice were cultured and expanded *ex vivo*, RNA was isolated from non-treated control (NT) and *C. perfringens* sialidase treated (CP) SFs, then subjected to bulk RNA-Seq (75bp paired-end, 30M reads, n = 3). **(A)** Principal component analysis (PCA). **(B)** All detected genes are plotted as a volcano plot. Genes that passed a threshold of padj < 0.01 and |foldChange| > 2 are considered differentially expressed genes (DEGs), comparing sialidase-treated and non-treated SFs. Colour code, red: upregulated, blue: downregulated, in CP treatment. **(C)** Upregulated genes in sialidase treatment identified in **(B)** were used to perform KEGG pathway enrichment analysis. KEGG pathways are plotted in bubble chart with fold enrichment on x-axis and -log10 p-value on the coloured scale. The size of bubble is proportional to the number of DEGs in the given pathway.

**Figure 4:**
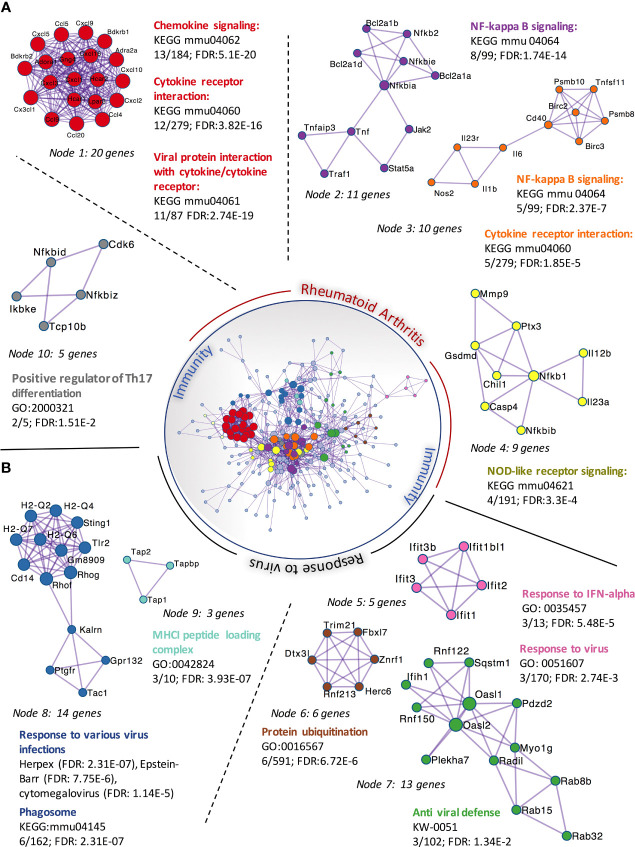
Protein-protein interaction networks of upregulated DEGs in *C. perfringens* sialidase treated SFs. Upregulated DEGs identified in **Figure 3** were used to perform protein-protein interaction enrichment in Metascape (https://metascape.org/ ). Genes are represented by coloured circles, size is directly proportional to the number of genes in each node. DEGs genes clustered a total of 10 independent nodes based on known protein-protein interactions. Functional pathways significantly represented in each node are shown. Nodes are classified into two main categories based on the functional roles: ‘rheumatoid arthritis’ **(A)** and ‘response to virus’ **(B)**.

We had previously observed that SFs up-regulated IL-6 and Ccl2 mRNA following loss of SA (26), but the functional consequences of SA down-regulation in SFs-mediated immunity were effectively unknown. Therefore, to identify SA-associated pathways and hence further understand the role of sialylation in SFs, non-treated control cells and cells desialylated with *C. perfringens* sialidase were subjected to RNA-Seq analysis. Principal Component Analysis identified that the two groups displayed distinct transcriptome profiles (**Figure 3A**). Thus, we searched for significant differential gene expression [DE fold change > 2, adjp <0.01] (**Figure 3B**) to identify distinct transcriptomic signatures associated with hyposialylated conditions. This DE gene list (**Supplementary Table 1**) was investigated for pathway enrichment using KEGG database (**Figure 3C**). Remarkably, SA removal induced a clear activated phenotype in SFs, including enriched pathways for Rheumatoid Arthritis, cytokine signaling (TNFα, IL-17, chemokines) and NFkB and TLR signalling (**Figure 3C**). Next, we used the MCODE algorithm *via* the bioinformatics tool Metascape (30) to find functional gene nodes among the differentially expressed genes in desialylated SFs, identifying 10 nodes able to hold significant interconnected protein interactions (**Figure 4**). All nodes could be grouped under the broad immunity label and were connected to a greater or lesser extent. Node 1 covered the larger number of genes, mostly related to the CXCL chemokine signalling and inflammatory mediators, indicating that SFs adopted a distinctive inflammatory stage upon desialylation. In line with this, nodes 2, 3 and 4 comprised genes involved in cytokine-cytokine receptor signalling, NOD-like receptor pathways and NFkB activation. Interestingly, pathways in nodes 1 to 4 resembled a typical SF response during RA (**Figure 4A**), even when cells in this experiment had not been stimulated with any inflammatory factor. In addition, another 5 functional nodes were defined [nodes 5-9], that were directly connected to pathways involved in responses to viruses or associated processes, such as interferon signalling, phagosome formation or MHC-I complexes (**Figure 4B**).

**Figure 5:**
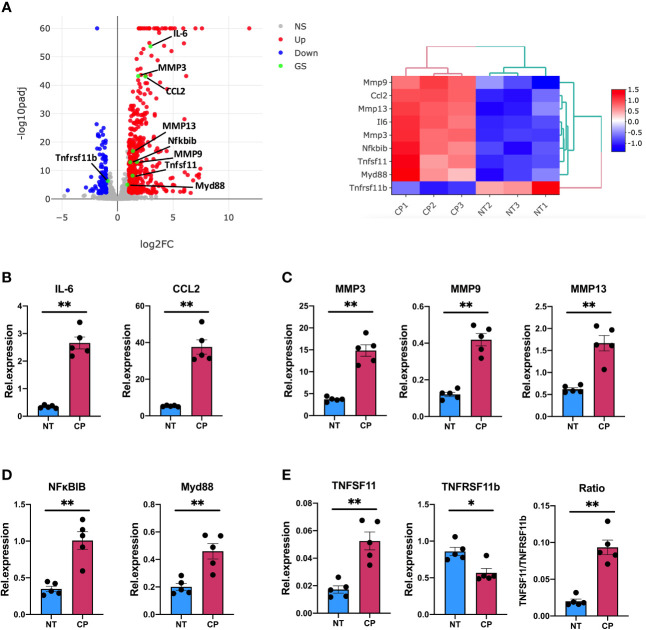
Sialidase-treated synovial fibroblasts show an enhanced inflammatory response. **(A)** mRNA expression as detected by RNA-Seq from **Figure 3** for MMP3, MMP9, MMP13, IL-6, CCL2, Myd88, Nfκbib, Tnfsf11 and Tnfrsf11b. **(B–E)** RNA was isolated from control (NT) and *C. perfringens* sialidase treated (CP) naïve SFs. Relative expression of the genes shown in **(A)** was assessed by RT-qPCR using the ΔΔCt method and actin as housekeeping gene. **(B)** IL-6 and CCL2, **(C)** MMP3, MMP9 and MMP13, **(D)** Myd88 and Nfκbib, and **(E)** Tnfsf11 and Tnfrsf11b. For **(B–E)**, each dot represents one independent experiment, error bars represent SEM (n = 5), *p < 0.05, **p < 0.01 evaluated by Mann-Whitney test. NS, non-significant.

Thus, our RNA-Seq data described in detail how elimination of sialic acid on SF surface acts as a molecular signal to activate an inflammatory and anti-viral programme. Intriguingly, such sialidase-induced inflammation showed common characteristics with the known inflammatory phenotype of activated SFs during chronic RA, albeit some other pathways were unrelated and resembled immune responses to viral infections. To corroborate the inflammatory capacity of desialylated SFs, we selected a representative set of genes associated with inflammatory joint disease whose expression was significantly enhanced in the RNA-Seq dataset to be evaluated in independent experiments by RT-PCR. Specifically, we selected 8 up-regulated genes representative of SF-mediated inflammation, including cytokines (IL-6, Ccl2), matrix metalloproteinases (MMP3, MMP9, MMP13) and NFkB signalling pathways (MyD88, NFκBIB). In addition, we evaluated TNFSF11 (TNFSF11, TNF Superfamily Member 11 or Receptor activator of nuclear factor kappa-B ligand, RANKL) and TNFRSF11B (TNF Receptor Superfamily Member 11b, or Osteoprotegerin, OPG) expression, because disturbed RANKL/OPG ratio promotes osteoclastogenesis and bone damage in RA (38). Corroborating RNA-Seq data (**Figure 5A**), IL-6, Ccl2, MyD88 and all MMPs were up-regulated in desialylated SFs (**Figures 5B-D**). Likewise, RANKL was up-regulated and OPG was down-regulated (**Figure 5E**), matching sequencing results and suggesting an increased osteoclastogenic potential in desialylated SFs.

**Figure 6:**
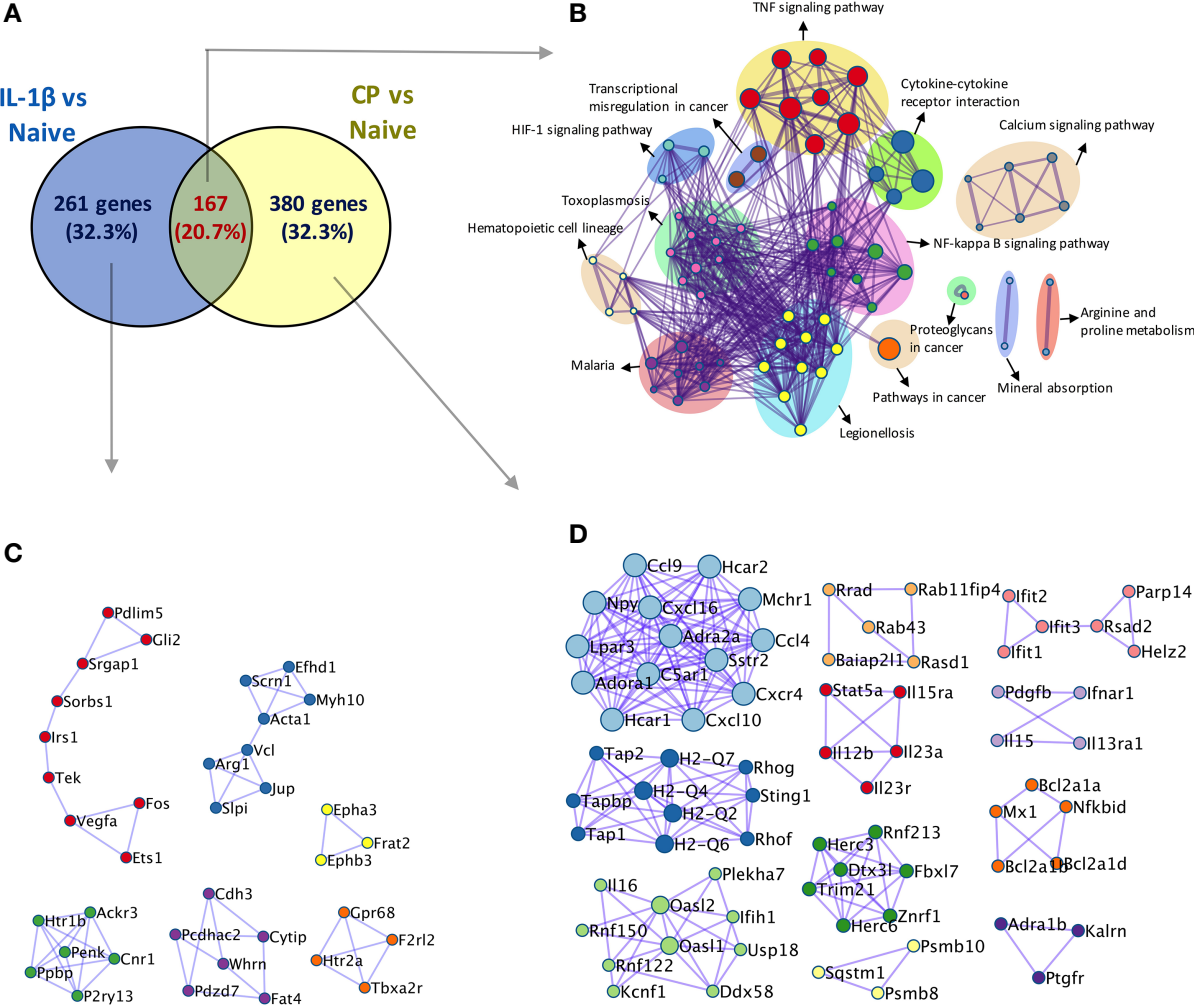
Comparison of transcriptomic activation of desialylated and IL-1β-stimulated SFs. Bulk RNA-Seq (75bp paired-end, 30M reads, n = 3) was performed on i) naïve and IL-1β-stimulated, and ii) non-treated and *C. perfringens*-treated naïve SFs, to identify DEGs (Padj < 0.01 and |foldchange| > 2) in both experimental conditions. **(A)** Venn diagram shows the number of overlapping and unique DEGs identified in the IL-1β vs naïve and CP vs NT comparisons. **(B)** Overlapping genes identified in **(A)** were used to perform pathway enrichment using the bioinformatics tool Metascape. **(C)** Unique genes in IL-1β vs naïve and **(D)** unique genes in CP vs NT identified in **(A)** were used to perform protein-protein interaction enrichment in Metascape.

Overall, our results show that hyposialylated SFs show an inflammatory phenotype reminiscent of arthritic cells, with an increased migratory ability and expression on pro-inflammatory cytokines and chemokines known to play a key role in RA. In fact, when RNA-Seq was used to compare SFs transcriptomic changes upon desialylation with those induced by IL-1β, a well-known inflammatory mediator in RA, we observed 20.7% of overlapping up-regulated genes between both experimental conditions (**Figure 6**). Among these genes, we found inflammatory cytokines and chemokines, like IL-6, Csf3, CXCL and Ccl members and MMPs (**Supplementary Table 2**), which showed significantly enriched pathways for cytokine-receptor signalling, NFκB signalling, and other immune pathways (**Figure 6**), further highlighting the pro-arthritic status of hyposialylated SFs.

### Enzymatic Removal of Surface SA Regulates Sialyltransferase Expression

**Figure 7:**
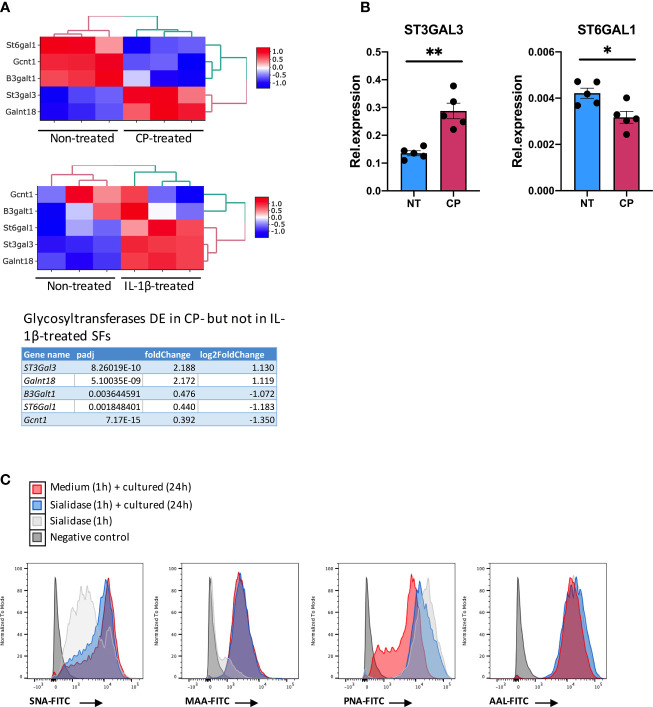
Enzymatic removal of sialic acid modulates intracellular sialyltransferases expression. **(A)** mRNA expression in SFs as detected by RNA-Seq from **Figure 6** for ST6Gal1, ST3Gal3, Gcnt1, B3Galt1 and Galnt18, including gene expression following *C. perfringens* sialidase treatment or IL-1β stimulation. Table shows adjp value and fold change of the glycosyltransferase genes, significantly regulated (adjp < 0.01, |log2Foldchange| >1) in *C. perfringens* sialidase treated SFs but not in in IL-1β-stimulated SFs. **(B)** RNA was extracted from control and *C. perfringens* sialidase treated naïve SFs. Relative expression of ST3gal3 and ST6gal1 was evaluated by RT-qPCR. Each dot represents one independent experiment, error bars represent SEM (n = 5), *p < 0.05, **p < 0.01 by Mann-Whitney test. **(C)** The presence of sialic acid on control and *C. perfringens* sialidase treated naive SFs was examined by flow cytometry for the binding of SNA, MAA, PNA and AAL. Experiments were performed after treatment (T0) and 24-hour incubation after treatment (T24).

Although the inflammatory signature of hyposialylated SFs shared a significant number of pathways with classical IL-1β stimulation, it also contained a distinctive set of DE 213 genes [fold change > 2, adjp <0.01] only observed in response to sialidase treatment (**Figure 6** and **Supplementary Table 2**). Among these, we still found a clear immune signature, including inflammatory cytokines (IL-15, IL-16), CXC chemokines and TNFα NF-κB signalling pathways. Cellular activation was evidenced by a large presence of P-loop NTPase fold-containing proteins, like Guanylate-binding proteins (Gbp), involved in the hydrolysis of phosphate bond of nucleoside triphosphates like ATP or GTP. This is mechanistically related to oxidative killing, phagolysosomes function and anti-viral responses, some of the other pathways activated only by desialylation. Interestingly, we also observed in our RNA-Seq datasets that the glycosyltranferase genes ST6Gal1, ST3Gal3, Gcnt1, B3Galt1 and Galnt18 were significantly regulated in response to surface desialylation, but not in response to IL-1β (**Figure 7A** and **Supplementary Table 2**). This suggests that exogenous factors inducing a loss of SA might provide a positive feedback to modulate endogenous expression of sialosides, which could represent a link between extracellular factors that modify SA content and consolidation of inflammatory response, perhaps leading to chronic disease. Hence, to evaluate whether SA removal also modulates SF-sialylation pathways, we analysed expression of the two sialyltransferases, St6Gal1 and ST3Gal3, by RT-PCR after *C. perfringens* sialidase treatment. Corroborating the RNA-Seq data, SF desialylation significantly down-regulated St6Gal1 mRNA expression, whilst it up-regulated ST3Gal3 mRNA expression (**Figure 7B**). Moreover, flow cytometry lectin binding experiments showed that CP-treated SFs had a decreased capacity to rebuild surface SA expression upon sialidase treatment, as evidenced by increased PNA-binding and decreased SNA-binding (**Figure 7C**). This could indicate that desialylation implements a molecular mechanism to maintain low levels of α2-6 sialylation even if the cells are no longer exposed to sialidase hydrolytic action.

## Discussion

In this study, we report that synovial fibroblasts undertake a highly inflammatory phenotype when SA is removed from the cell surface. This supports our *in vivo* results, showing that inflammatory SFs show lower sialylation than healthy cells, mainly α2-6 linked SA. Notably, sialylation has been involved in cellular processes that are critical for SFs-mediated pathophysiology in RA, like cell migration and immune regulation. SA also determines metastatic potential and migration in cancer as well as tumour aggressiveness and invasiveness (39, 40). Specifically, elevated α2-3 sialylation has been associated with enhanced migration in breast cancer, melanoma and pancreatic adenocarcinoma (41–43). Likewise, pathogenic cell migration has also been reported for α2-6 linked SA. Pally et al. described that distinct sialylation levels correlate with migratory phenotypes of epithelial cancer cells in three-dimensional cultures (44) and endometriotic cells show enhanced migration after α2-6 desialylation (45). Similar mechanisms could therefore happen in the arthritic joint to promote SFs migration and invasion of cartilage tissue. However, local factors such as the composition of the extracellular matrix in health and disease will influence cell migration. Thus, comparisons with other cell types or environments should be taken with caution and tailored studies are needed to fully understand how SA might affect SF migration in the context of arthritis.

Our data also revealed a strong link between low sialic acid content and initiation of SFs inflammatory and immune responses, suggesting that sialic acid acts as a molecular switch to control tissue homeostasis and inflammation in the joint synovial space. Our *in vivo* data show that a decreased α2-6/α2-3 SA ratio is responsible of SFs activation, something that *C. perfringens* sialidase recapitulates *in vitro*, proving that hyposialylated SFs become highly inflammatory. In this regard, the pathophysiology of SA in cancer represents the opposite scenario to autoimmune RA, as high SA content provides an advantageous scenario to cancer cells, particularly from the perspective of moderating immune responses. Tumour cells evade immune responses by adopting a hypersialylated phenotype to exploit SA-Siglec immunosuppressive signalling (46–49), whereas highly sialylated SFs may provide the immunosuppressive environment required in joint physiology. These overall results offer strong support to consider SA as an immunoregulatory switch, whose opposite actions in cancer and autoimmunity may represent two sides of the same coin. Considering this, SA is a potential target in cancer immunotherapy, since eliminating SA restraint in the tumour microenvironment could release anti-tumour immunity. Early studies dating back a few decades showed that treatment of a leukaemia cell lines with neuraminidases increased their immunogenicity (50, 51). A more specific approach is the recent development of engineered antibody–sialidases conjugates to target Siglec-dependent binding of NK cells, which makes tumour cells more susceptible to antibody-dependent cell-mediated cytotoxicity (52). However, our results in SFs could perhaps suggest that biologics with sialidase activity could also have the potential of starting off-target inflammation and autoimmunity, although additional work in animal models or clinical studies are required to challenge this hypothesis. Nevertheless, further steps have already been taken to optimise the selectivity of antibody-sialidases conjugates, by assessing several recombinant sialidases (53), but consideration of sialidase-dependent immune effects in long-term therapeutic regimes may be carefully considered for the development of safer and better sialidase-conjugated biologics.

Our study provides a causal link between presence of SA and initiation of immune responses in stromal SFs, but it still presents important limitations that should be addressed in follow-up studies. We used a recombinant sialidase from *C. perfringens* but details about the specific changes in sialylated glycoconjugates are still unknown. Specific SA linkages, membrane distribution, and SA acetylation might be an important aspect of SA-dependent communication and signalling. In fact, studies using CRISPR Cas9 showed that SA acetylation affects Siglec-mediated functions (54). Besides, the predominant sialic acids on murine cells are N-acetylneuraminic acid (Neu5Ac) and N-glycolylneuraminic acid (Neu5Gc), the latter not synthesised in humans because of the loss of the CMAH gene. This evolutionary event caused a rapid adaptation of the Siglec family to the new human glycome dominated by Neu5Ac. Therefore, translation of findings from murine models to human biology may be challenging, especially given that several pathotypes have been described in human RA (55). It is still very unclear how inflammatory mediators modulate the content of SA in human RA to initiate or perpetuate inflammation, and further studies, considering disease heterogeneity, should be conducted. To facilitate these translational findings, it is required to delineate the molecular mechanisms underlying SA-dependent SF activation. Loss of SA would imply a lack of regulatory Siglec signalling, but it may also uncover underlying galactose residues allowing galectin-3-binding and subsequent cell activation, as galectin-3 is a highly inflammatory mediator. In fact, galectin-3 induces a higher expression of pro-inflammatory IL-6, GM-CSF, MMP3 and even TNFα in SFs than in skin fibroblasts (3). A reduction of such inflammatory mediators in the synovium would also reduce local inflammation and cell recruitment, further reducing local TNFα and maintaining high levels of sialylation, since inflammatory TNFα down-regulates ST6Gal1 expression and α2-6 sialylation (26). Therefore, the inflammatory axis TNFα-hyposialylation-galectin-3 could have a stronger impact in the synovium compared with other tissues, such as the skin, perhaps explaining the tissue tropism observed in RA inflammation. Nevertheless, the potential effect of hyposialylated fibroblasts must also be investigated in other tissue more prone to suffer chronic inflammation, like the lung or gut. Interestingly, low sialylation has been linked to other pathways and cell types during RA, for example, activated chondrocytes show reduced levels of α2-3 SA and hyposialylation is also observed in Rheumatoid arthritis (RA)-associated IgG antibodies (56, 57).

Finally, we believe that understanding the factor(s) responsible of SF loss of SA *in vivo* is of high relevance to understand chronic RA. Such factors could have heterogeneous origins including i) cytokine signalling, like the TNFα-mediated downregulation of ST6Gal1 as we have shown before, ii) endogenous sialidases, secreted by SFs or other immune cells in arthritic joint, iii) infections, for example viral sialidases and iv) environmental factors, like diet and environment. For example, cigarette smoke reduces ST6Gal1 and α2-6 sialylation in bronchial epithelial cells leading to IL-6 production (58), similarly to the effects that we have observed in SFs upon desialylation. Similarly, sialidases are secreted by several viruses and other pathogens to modulate SA-dependent actions (59, 60), perhaps indicating that infections can remodel the local glycome to trigger, or favour, the establishment of future inflammatory RA. The fact that desialylated SFs activate anti-viral responses would provide support to such viral infection-chronic inflammation link. Importantly, our results show that removal of surface SA reconfigures expression of some sialyltransferases, suggesting that initial SA loss can lead to chronic inflammatory feedbacks, contributing to perpetuation of disease in RA regardless of the initiating factors.

## Data Availability Statement

The data presented in this study are deposited in NCBI's Gene Expression Omnibus and are accessible through GEO Series accession numbers GSE192488 and GSE196898.

## Ethics Statement

The animal study was reviewed and approved by Ethics Review Board of the University of Glasgow.

## Author Contributions

MP conceived and performed experiments, oversaw the project, interpreted the results, and wrote the manuscript with feedback from all authors. YW performed experiments and contributed to design of the experiments, data analysis and manuscript writing. PP, CC, and AK performed experiments. All authors contributed to the article and approved the submitted version.

## Funding

The work was funded by a Career Development award to MP from Versus Arthritis (21221), a China Scholarship Council (CSC) PhD scholarship awarded to YW and a YLSY Turkish Ministry of National Education Study Abroad Programme awarded to CC.

## Conflict of Interest

The authors declare that the research was conducted in the absence of any commercial or financial relationships that could be construed as a potential conflict of interest.

## Publisher’s Note

All claims expressed in this article are solely those of the authors and do not necessarily represent those of their affiliated organizations, or those of the publisher, the editors and the reviewers. Any product that may be evaluated in this article, or claim that may be made by its manufacturer, is not guaranteed or endorsed by the publisher.
